# Global burden, trends, and disparities in kidney cancer attributable to smoking from 1990 to 2021

**DOI:** 10.3389/fpubh.2024.1506542

**Published:** 2025-01-08

**Authors:** Siyu Han, Shiyu Zhao, Ran Zhong, Peizhe Li, Yuewen Pang, Shuang He, Junyao Duan, Huijie Gong, Jing Shi, Li Liu, Yongji Yan

**Affiliations:** ^1^Department of Urology, Dongzhimen Hospital, Beijing University of Chinese Medicine, Beijing, China; ^2^Shanghai General Hospital, Shanghai Jiao Tong University School of Medicine, Shanghai, China

**Keywords:** burden of disease, kidney cancer, smoking, death, disability-adjusted life-years

## Abstract

**Purpose:**

Smoking is a well-established risk factor for kidney cancer. Analyzing the latest global spatio-temporal trends in the kidney cancer burden attributable to smoking is critical for informing effective public health policies.

**Methods:**

Using data from the 2021 GBD database, we examined deaths, disability-adjusted life years (DALYs), and age-standardized rate (ASR) of kidney cancer attributable to smoking across global, regional, and national levels. Trends in ASRs were assessed through estimated annual percentage change (EAPC). We conducted a cross-country analysis to evaluate disparities in the kidney cancer burden from 1990 to 2021, with absolute and relative inequalities measured by the slope index of inequality and concentration index, respectively. Correlation analysis was conducted by the Spearman rank order correlation method. Additionally, we projected age-standardized death and DALYs rates up to 2036 using Bayesian age-period-cohort (BAPC) models in R.

**Results:**

Globally, kidney cancer deaths attributable to smoking increased by 67.64%, from 9,673 in 1990 to 16,216 in 2021. Despite this increase, the age-standardized death rate (ASDR) dropped from 0.25 to 0.19 per 100,000 (EAPC: −0.93). Similarly, the age-standardized disability-adjusted life years rate (ASDALY) decreased from 6.17 to 4.37 per 100,000 (EAPC: −1.15). Geographically, areas with a higher Socio-demographic Index (SDI) were the most affected. The positive correlation between higher SDI and increased deaths highlights the role of economic and social factors in disease prevalence. Cross-country analysis shows that while relative inequalities between groups are improving, absolute differences in health burdens continue to grow. Furthermore, projections indicate a gradual decline in ASDR and ASDALY for both sexes from 2022 to 2036.

**Conclusion:**

Between 1990 and 2021, both the global ASDR and ASDALY attributable to smoking in kidney cancer, which are positively correlated with SDI, have declined. However, significant demographic and geographic disparities persist, with the disease burden remaining higher in older populations and regions with elevated SDI levels. Moreover, while the overall burden is projected to decline annually over the next 15 years, it is expected to remain significantly higher in men. These findings emphasize the need for region-specific health prevention strategies to reduce smoking-related kidney cancer.

## Introduction

Kidney cancer, one of the top ten cancer-related killers, is a common malignancy of the genitourinary system and is associated with a poor prognosis. It remains difficult to detect, lacks public awareness, and presents challenges in treatment (1), all of which place a heavy financial burden on individuals and families. Even survivors often face economic hardship and psychological stress (2). Renal cell carcinoma (RCC) accounts for over 90% of kidney cancers (3). According to the latest global epidemiological study published by the European Renal Association, the incidence of kidney cancer is rising, with approximately 400,000 new cases and nearly 175,000 deaths annually (4). This increase is closely linked to global population growth and aging. However, significant geographic and temporal variations exist, leading to differences in incidence rates across regions (5).

Evidence from the GBD 2021 Risk Factors Collaborators identified smoking as the third leading risk factor for the global burden of disease across all ages (6). For RCC, tobacco smoking, obesity, and hypertension are well-established risk factors (3). Over the past several decades, substantial epidemiological evidence has demonstrated a strong association between tobacco use and various human cancers. A 2004 report by the United States Surgeon General established a direct causal link between tobacco use and kidney cancer (7). Additionally, the International Agency for Research on Cancer classified smoking as a kidney carcinogen, with sufficient evidence supporting a causal relationship between smoking and RCC (8, 9). Several studies have also reported positive associations between smoking and kidney cancer incidence or mortality, with both current and former smokers exhibiting an elevated risk even after adjusting for body mass index and hypertension (10–13). A meta-analysis indicated that ever-smokers have a relative risk of 1.38 for RCC (14), while smoking cessation shows a protective effect in never-smokers and those who quit years prior (15).

Furthermore, studies have demonstrated associations between secondhand smoke, chewing tobacco, and the development of kidney cancer. Specifically, a Health and Nutrition Examination Survey conducted in the United States revealed that exposure to secondhand smoke significantly increases the risk of developing kidney cancer (16). Similarly, a US case–control study from 1977 to 1993 found that in patients with RCC, the odds ratio (OR) for developing kidney cancer due to chewing tobacco was 3.2 (95% CI, 1.1–8.7) (17), further reinforcing the link between tobacco use and kidney cancer.

Understanding the burden of kidney cancer due to smoking across different regions is essential for developing effective tobacco control policies and improving health outcomes. Previous research on kidney cancer has primarily focused on the overall disease burden from all risk factors, resulting in a lack of detailed analysis on the specific burden attributed to smoking and the implementation of targeted preventive measures (18–20). In this study, we analyzed data from GBD 2021, combining factors like year, gender, age, and sociodemographic index (SDI), to examine current trends in smoking-related kidney cancer deaths and disability-adjusted life years (DALYs) at global, regional, and national levels.

## Materials and methods

### Data resource and definitions

This study used data from the GBD 2021 study (https://vizhub.healthdata.org/), focused on the burden of kidney cancer due to smoking from 1990 to 2021 across 204 countries and territories within 21 GBD regions, further categorized into five groups based on the SDI. We concentrated on two primary metrics: deaths and DALYs. Kidney cancer was defined according to ICD-10 codes C64-C65 (21). The term ‘smoking’ is defined as current daily or occasional use of any smoked tobacco product (6). DALYs represent the total number of years lost due to premature death and the number of years lived with disability. The SDI provides a comprehensive assessment of the development level of each country and region by combining three indicators: per capita income, fertility rate, and education level. It ranges from 0 to 1, with 1 representing the highest level of development and 0 the lowest (21).

### Measurement health inequalities

As recommended by the World Health Organization, we used the Slope Index of Inequality (SII) and the Concentration Index (CI) to assess absolute and relative income-related inequality among countries (22). The SII was calculated using regression analysis, which compares the national DALYs rate for all age groups to an SDI-based relative location scale. The heteroscedasticity was treated with a weighted regression model. Higher absolute SII values indicate greater inequality. The CI, ranging from-1 to 1, was used to evaluate relative disparities in kidney cancer burden attributable to smoking across countries by fitting a Lorenz curve based on cumulative DALYs and population. A negative CI value indicates a higher concentration of the kidney cancer burden due to smoking among populations residing in countries with lower SDI.

### Statistical analysis

To ensure the comparability of statistical indicators, we used the age-standardized death rate (ASDR), age-standardized disability-adjusted life years rate (ASDALY), and their corresponding 95% uncertainty interval (95% UI) to assess and compare kidney cancer deaths and DALYs at both regional and national levels (21).

To assess temporal changes in the age-standardized rates (ASR) of kidney cancer deaths and DALYs attributable to smoking from 1990 to 2021, we employed the Estimated Annual Percentage Change (EAPC). The regression model of ASR over time is as following equation: ASR = *α* + *β*x + *ε*, where β represents the regression coefficient, x is the calendar year, and ε is the error term. The EAPC and its 95% confidence interval (95% CI) were obtained via the formula EAPC = 100 × (exp(β) − 1).

If the lower bound of the 95% CI for EAPC is greater than zero, it indicates an increase in ASR. Conversely, if the upper bound of the 95% CI is less than zero, it suggests a decline. If neither condition is met, the ASR is considered stable. Data analysis was conducted using R (version 4.3.3), with the ggplot2 and RColorBrewer packages used for data visualization. Spearman’s rank-order correlation analysis was conducted to investigate the associations between SDI and both ASDR and ASDALY. Statistical significance was defined by a *p*-value below 0.05. Bayesian Age-Period-Cohort (BAPC) models, combined with integrated nested Laplacian approximation (INLA), were used to estimate and project death and DALYs rates for males and females through 2036. The analysis was performed using the BAPC and INLA packages in R (23).

## Results

### Global burden

The number of deaths and DALYs from kidney cancer attributable to smoking increased globally between 1990 and 2021. Smoking-related kidney cancer deaths rose from 9,673 (95% UI: 6,072 to 13,617) in 1990 to 16,216 (95% UI: 9,663 to 23,217) in 2021, reflecting a 67.64% increase. Similarly, global DALYs for kidney cancer due to smoking increased from 251,336 (95% UI: 159,401 to 348,868) in 1990 to 382,927 (95% UI: 233,635 to 536,755) in 2021 (Supplementary Table S1).

### Burden by SDI

**Figure 1:**
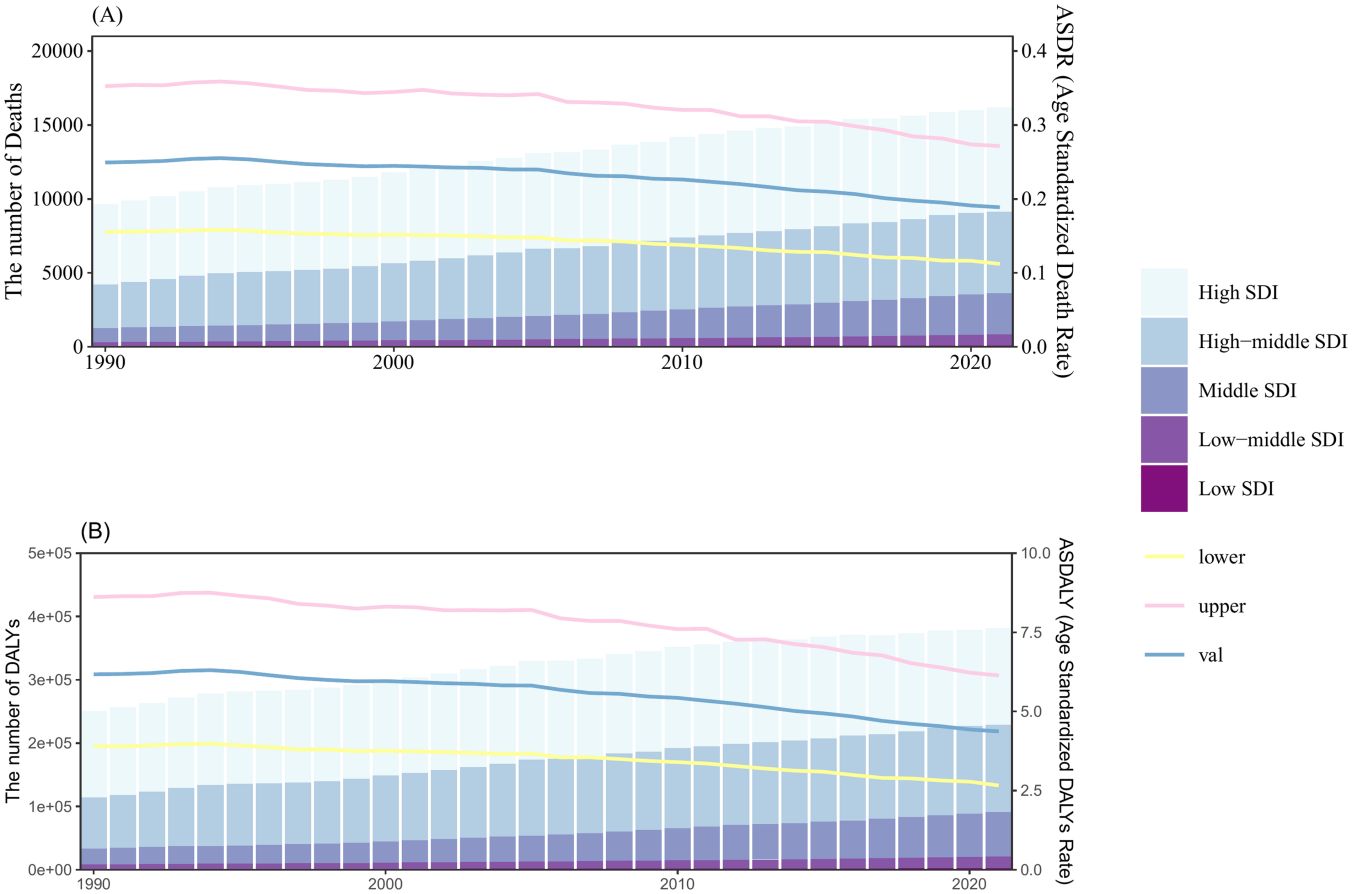
Number and rate of kidney cancer deaths **(A)** and DALYs (B) attributable to smoking between 1990 and 2021, according to the SDI. The bar chart shows the number of smoking-related kidney cancer deaths (A) and DALYs (B), categorized by SDI levels. The line represents the global mean ASDR (A) and ASDALY **(B)** (per 100,000) attributable to smoking. The shaded area represents the 95% UI for the mean rate. Results are shown for both sexes at the global level. DALYs, disability-adjusted life-years; ASDR, age-standardized death rate; ASDALY, age-standardized DALYs rate; SDI, socio-demographic index; UI, uncertainty interval.

Data analysis across the five SDI regions showed a general year-by-year increase in both deaths and DALYs attributable to smoking-related kidney cancer. The distribution of deaths and DALYs was closely aligned with SDI levels, with higher SDI regions experiencing greater numbers of deaths and DALYs. In 2021, the high SDI region recorded 7,046 deaths (95% UI: 4,030 to 10,669), the middle SDI region had 2,802 deaths (95% UI: 1,718 to 3,871), and the low SDI region reported 101 deaths (95% UI: 56 to 151). The highest ASDR and ASDALY values were found in the high SDI region, while the lowest were in the low SDI region. Globally, ASDR and ASDALY have been steadily declining over the years (Figure 1; Supplementary Table S1).

**Figure 2:**
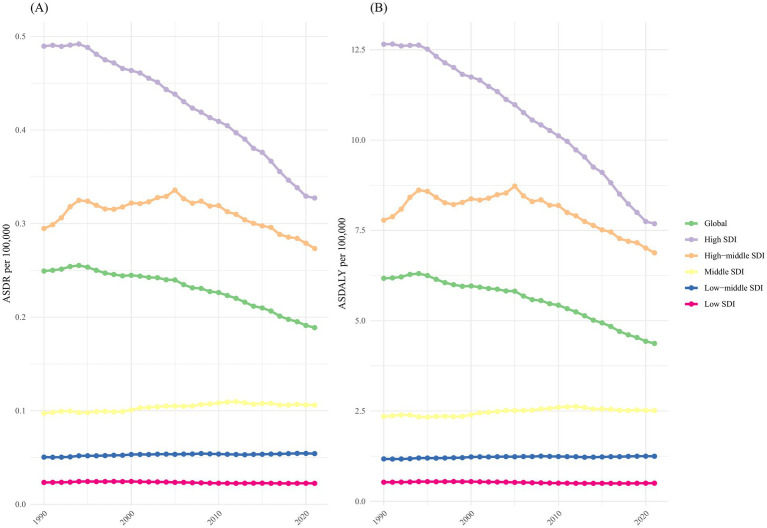
The burden of kidney cancer attributable to smoking by SDI. The ASDR **(A)** and ASDALY **(B)** of kidney cancer attributable smoking in different SDI regions from 1990 to 2021. Results are shown for both sexes at the global level. DALYs, disability-adjusted life-years; ASDR, age-standardized death rate; ASDALY, age-standardized DALYs rate; SDI, socio-demographic index.

As depicted in Figure 2, the ASDR and ASDALY of kidney cancer attributable to smoking in the high and high-middle SDI regions exhibited a consistent declining trend from 1990 to 2021, while other SDI regions remained relatively unchanged. Regions with higher SDI values, such as high and high-middle SDI, experienced a greater burden of ASDR and ASDALY compared to the global average. In contrast, regions with lower SDI values had lower burdens and ASDR and ASDALY than the global average (Figure 2).

### Burden by geographic region

Among the 21 geographic regions, East Asia recorded the highest absolute numbers of kidney cancer-related deaths and DALYs attributable to smoking in 2021, with 3,614 deaths (95% UI: 2,156 to 5,143) and 90,535 DALYs (95% UI: 53,869 to 127,788). In contrast, Oceania had the lowest absolute numbers, with just 1 death (95% UI: 1 to 2) and 40 DALYs (95% UI: 18 to 68) (Supplementary Table S1). Central Europe had the highest ASDR [0.49 (95% UI: 0.3 to 0.71)], while Eastern Europe had the highest ASDALY [12.81 (95% UI: 8.22 to 17.74)]. The lowest ASDR and ASDALY were observed in Western Sub-Saharan Africa, with an ASDR of 0.01 (95% UI: 0.01 to 0.01) and an ASDALY of 0.22 (95% UI: 0.12 to 0.34) (Supplementary Table S1).

**Figure 3:**
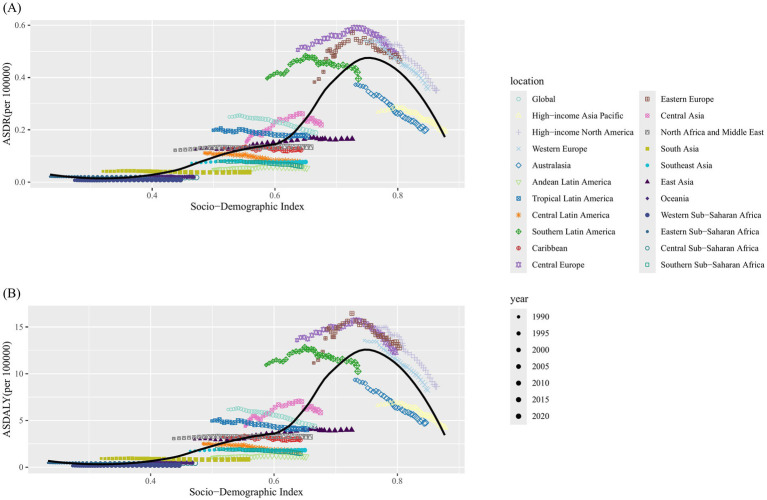
Correlations between ASDR and ASDALY of kidney cancer attributable to smoking and SDI at the regional level. The ASDR **(A)** and ASDALY **(B)** of kidney cancer attributable to smoking in 21 regions from 1990 to 2021. ASDR, age-standardized death rate; ASDALY, age-standardized DALYs rate; SDI, socio-demographic index.

Correlation analysis was conducted to examine the relationship between SDI and the 21 regions in terms of ASDR and ASDALY of kidney cancer attributable to smoking. Our findings showed that higher SDI levels were generally associated with higher ASDR (*R* = 0.85, *p* < 0.001) and ASDALY (*R* = 0.84, *p* < 0.001); However, no linear associations were identified for either measure (Figure 3). At the regional level, Eastern Europe, Central Europe, Western Europe, and High-income North America exhibited higher ASDR than expected based on their SDI values. In contrast, Australasia, Andean Latin America, and Southern Sub-Saharan Africa showed lower ASDR than anticipated for their SDI levels (Figure 3A). A similar pattern was observed for ASDALY in these regions (Figure 3B).

**Figure 4:**
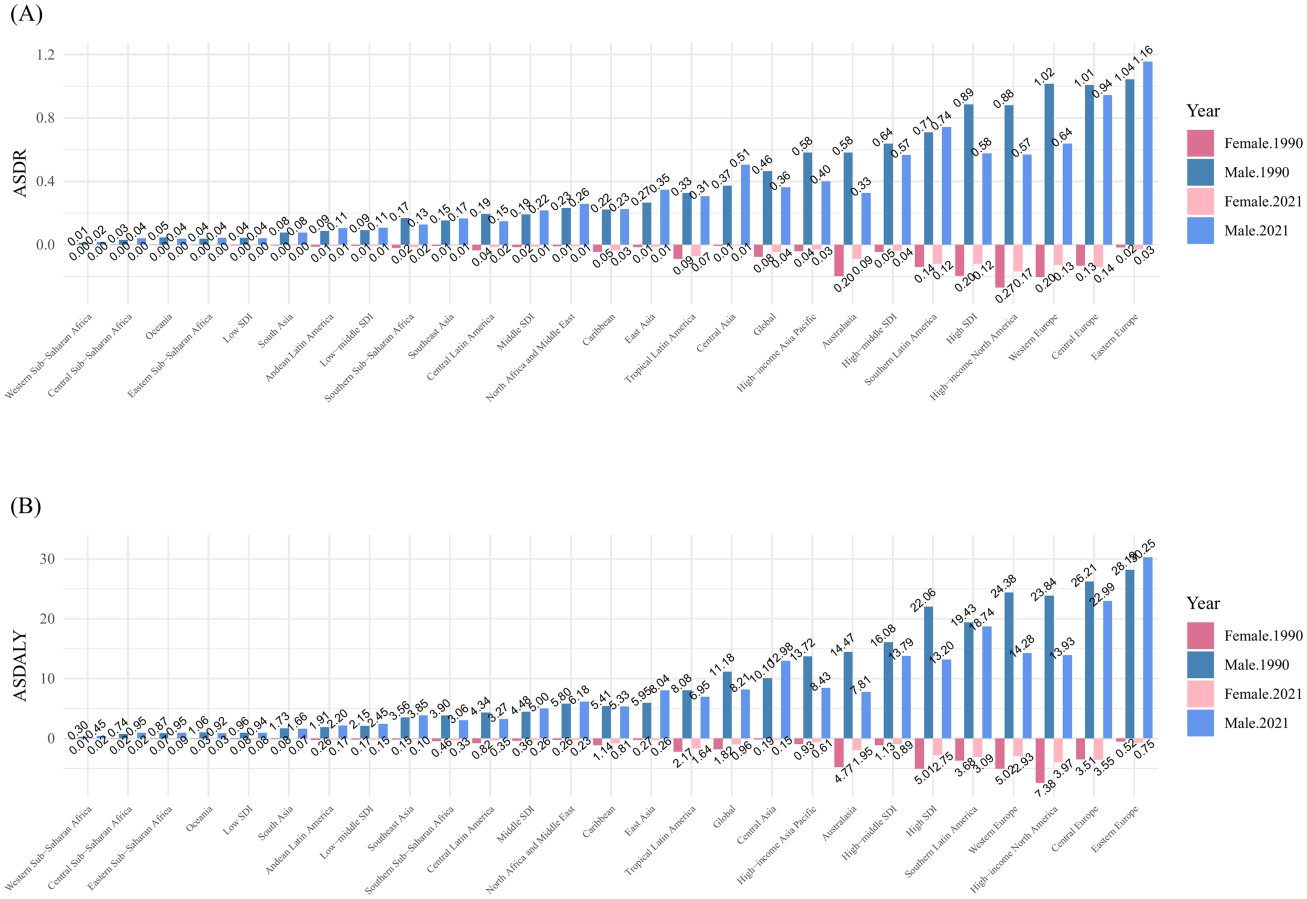
Distribution of ASDR **(A)** and ASDALY **(B)** for kidney cancer attributable to smoking, by sex, in 1990 and 2021, globally and across 21 geographic regions and SDI regions.

The ASDR and ASDALY for 1990 and 2021 by sex are compared across various regions, with the values arranged in ascending order from left to right. The comparison shows a significant gender disparity in the burden of kidney cancer due to smoking. In 2021, the ASDR for males [0.36 (95% UI: 0.22 to 0.52)] was approximately 9 times higher than that for females [0.04 (95% UI: 0.03 to 0.07)], and the ASDALY for males [8.21 (95% UI: 5.01 to 11.50)] was about 8.5 times higher than that for females [0.96 (95% UI: 0.57 to 1.43)] (Figure 4).

### Burden by country

**Figure 5:**
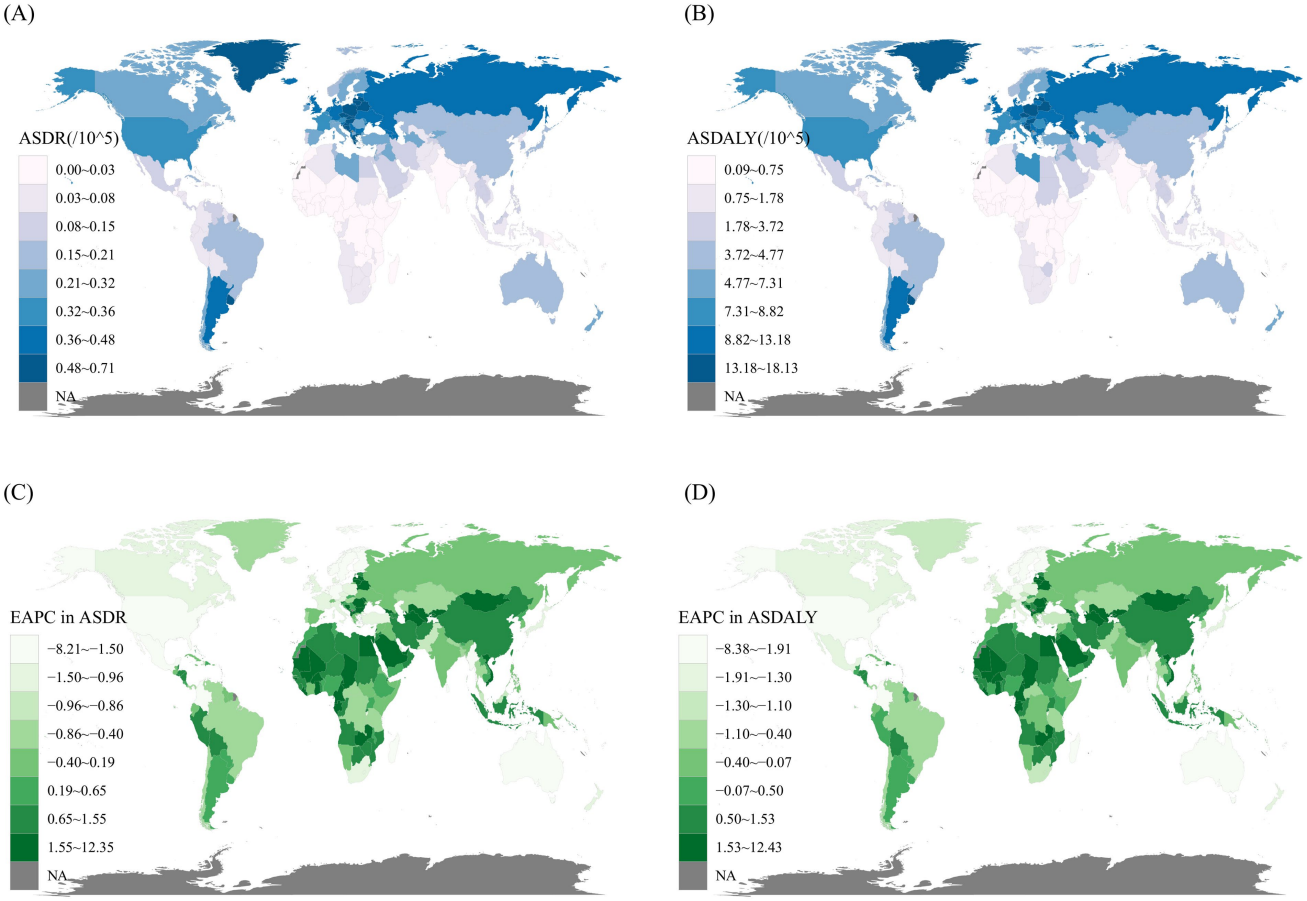
The spatial distribution of kidney cancer ASDR **(A)** and ASDALY **(B)** attributable to smoking in 2021, along with the EAPC in kidney cancer ASDR **(C)** and ASDALY **(D)** attributable to smoking. ASDR, age-standardized death rate; ASDALY, age-standardized DALYs rate; EAPC, estimated annual percentage change.

From the perspective of GBD regions, China and the United States had the highest number of kidney cancer-related deaths attributable to smoking in 2021, with 3,481 deaths (95% UI: 2,068 to 4,957) and 2,128 deaths (95% UI: 1,143 to 3,337), respectively. Following closely were Russian Federation, Germany, and Japan. A similar trend was observed for DALYs, with China recording 87,271 cases (95% UI: 51,675 to 124,166), the United States 50,007 cases (95% UI: 28,671 to 76,466), and the Russian Federation 31,699 cases (95% UI: 20,501 to 43,854), ranking as the top three countries. In terms of ASDR and ASDALY, Uruguay [ASDR: 0.71 per 100,000 (95% UI: 0.41 to 1.06), ASDALY: 18.13 per 100,000 (95% UI: 10.82 to 26.34)], Czechia [ASDR: 0.69 per 100,000 (95% UI: 0.4 to 1.04), ASDALY: 16.69 per 100,000 (95% UI: 10.1 to 25.07)], and Greenland [ASDR: 0.68 per 100,000 (95% UI: 0.36 to 1.08), ASDALY: 16.46 per 100,000 (95% UI: 8.94 to 26)] were the top three countries (Figures 5A, B).

Between 1990 and 2021, the rates of change in ASDR and ASDALY varied significantly across 204 countries. The countries with the highest increases in the EAPC of ASDR was Mongolia [12.35 (95% CI: 10.81 to 13.92)], followed by the Republic of Cabo Verde [9.26 (95% CI: 6.62 to 11.97)] and Turkmenistan [7.03 (95% CI: 5.48 to 8.59)]. The ASDALY trends mirrored this pattern. Conversely, the countries with the largest decreases in both ASDR and ASDALY were the Democratic Socialist Republic of Sri Lanka, the Kingdom of Norway, and the Republic of Fiji (Figures 5C, D).

### Burden by age

**Figure 6:**
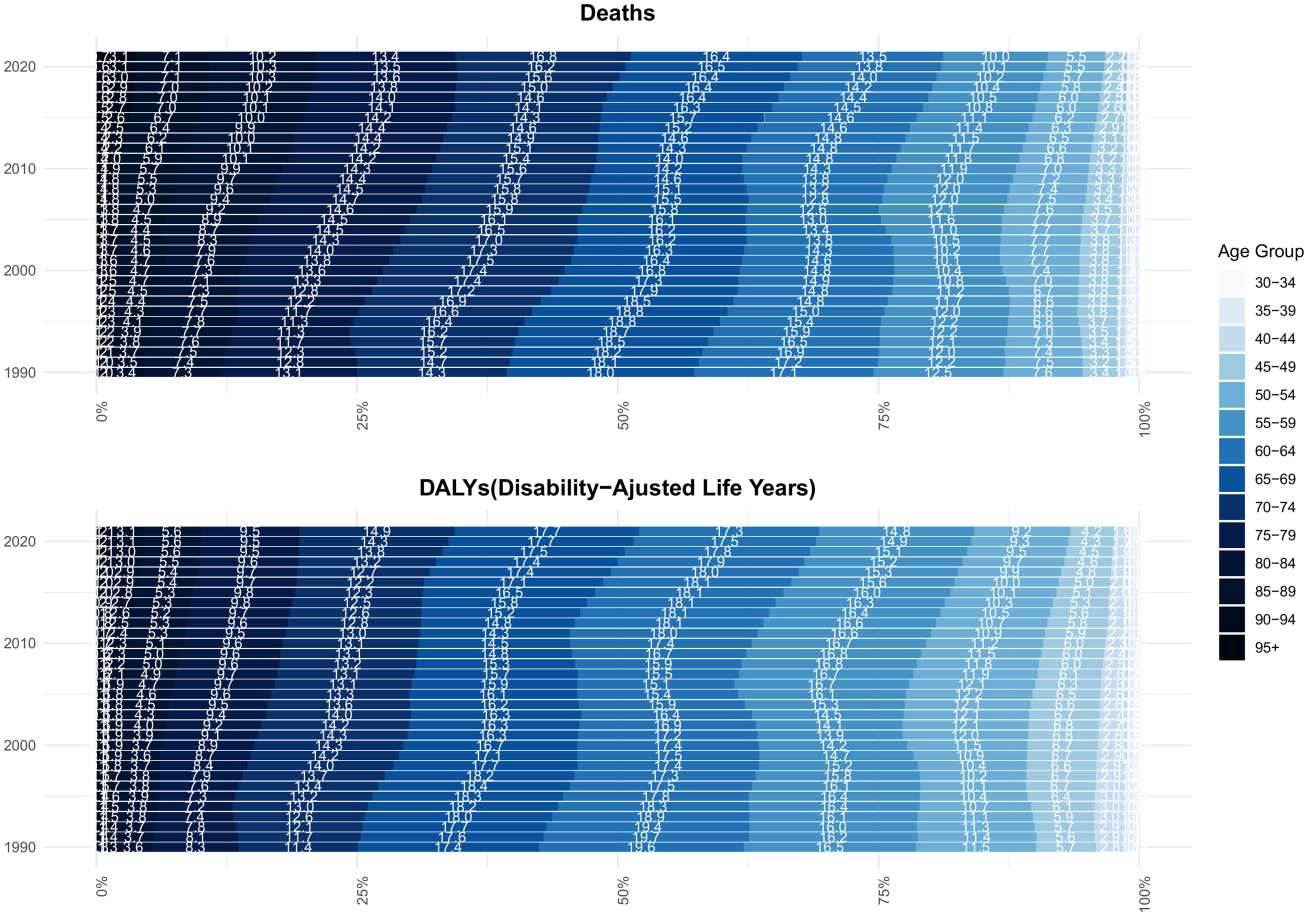
Age composition ratio of kidney cancer deaths and DALYs attributable to smoking, 1990–2021. DALYs: disability-adjusted life-years.

**Figure 7:**
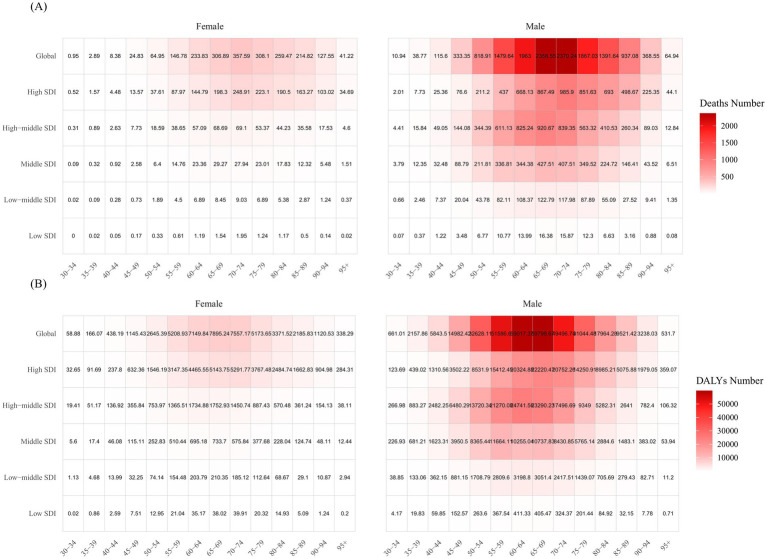
Kidney cancer deaths **(A)** and DALYs **(B)** attributable to smoking, by age group, gender, and SDI region in 2021.

In 2021, individuals aged 65 and older accounted for 67.7% of total deaths, a notable rise from 57.3% in 1990. Back in 1990, the 65–69 age group held the largest share of deaths at 18%, but by 2021, this shifted to the 70–74 age group, which accounted for 16.8%. As for DALYs, the 60–64 age group represented the highest proportion in 1990 at 19.6%, while in 2021, the 65–69 age group contributed the most, at 17.7% (Figure 6). In 2021, the highest number of kidney cancer deaths attributable to smoking occurred in both men and women aged 70–74 years (Figure 7A), whereas the greatest burden of DALYs was seen in the 65–69 age group (Figure 7B). Notably, both the number of deaths and DALYs were lower in women compared to men. The disease burden was significantly higher in regions with elevated SDI levels (Figure 7).

**Figure 8:**
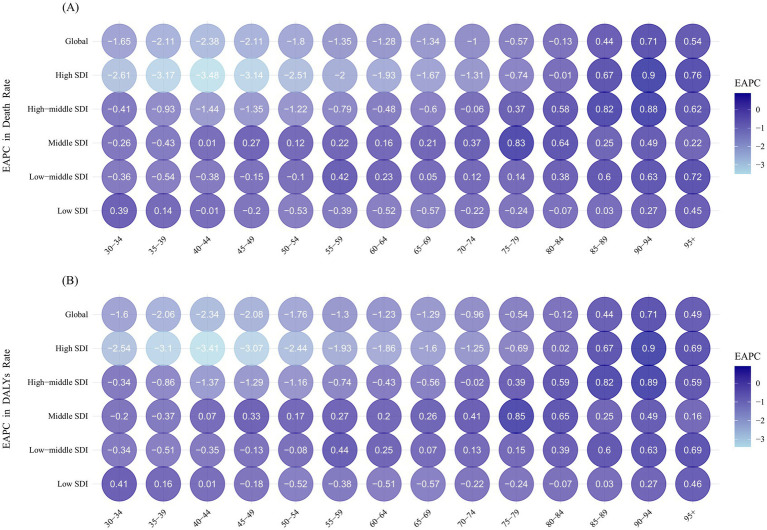
Estimated annual percentage change in death **(A)** and DALYs **(B)** rates between 1990 and 2021, by age group and SDI region. DALYs, disability-adjusted life years; SDI, socio-demographic index.

The EAPC for individuals aged 85 and older was consistently greater than zero, both globally and across all SDI regions, except for the low SDI region. However, trends in ASR varied significantly for age groups under 85 years across different SDI regions. Globally, ASR in the 85+ age groups increased, while those in age groups under 80 years showed a decline. The high SDI region followed this global pattern, with ASRs in younger age groups declining at a faster rate. For example, in the 35–39 age group, the EAPC for ASDR was −3.17 (95% CI: −3.33 to −3.01) and for ASDALY, −3.10 (95% CI: −3.26 to −2.94). Similarly, in the 40–44 age group, the EAPC for ASDR was −3.48 (95% CI: −3.72 to −3.25) and for ASDALY, −3.41 (95% CI: −3.65 to −3.17) (Figure 8A,B; Supplementary Tables S2, S3). In the middle and low-middle SDI regions, ASRs across different age groups generally increased, while in the low SDI region, most age groups experienced a decrease in ASR (Figures 8A,B).

### Cross-country inequalities

**Figure 9:**
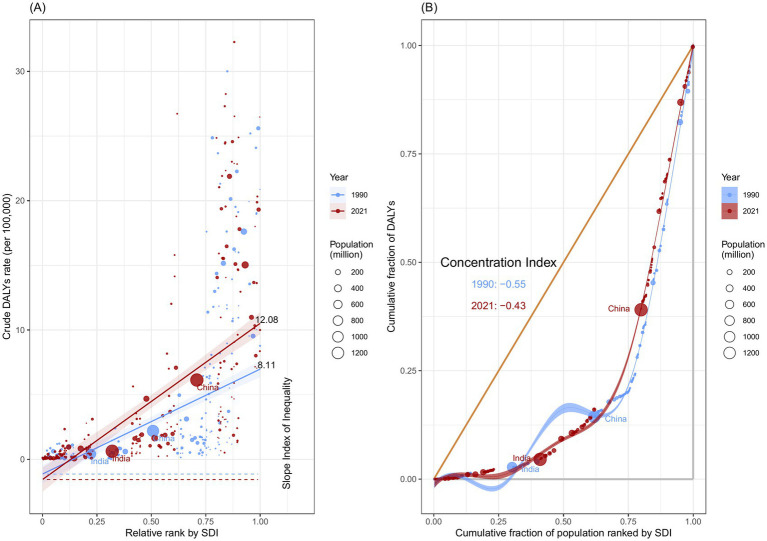
Health inequality regression curves **(A)** and concentration curves **(B)** for the DALYs of kidney cancer attributable to smoking, across 204 counties and territories, 1990 and 2021.

In 1990, the SII for DALYs (per 100,000 population) was 8.11 (95% CI: 6.68 to 9.55), increasing to 12.08 (95% CI: 10.17 to 13.99) in 2021. This indicates that DALYs rates were consistently higher in countries with higher SDI values in both years, and that inequality has increased over time (Figure 9A).

The results of the CI showed that between-country inequality in the distribution of kidney cancer burden attributable to smoking decreased from −0.55 (95% CI: −0.63 to −0.47) in 1990 to −0.43 (95% CI: −0.52 to −0.34) in 2021. This indicates that, while the burden of disease remains concentrated in low SDI countries, the degree of concentration has lessened over time (Figure 9B). Despite the narrowing wealth gap in certain regions, global inequality in kidney cancer attributable to smoking continues to be a persistent issue.

### Global disease burden prediction for kidney cancer attributable to smoking

**Figure 10:**
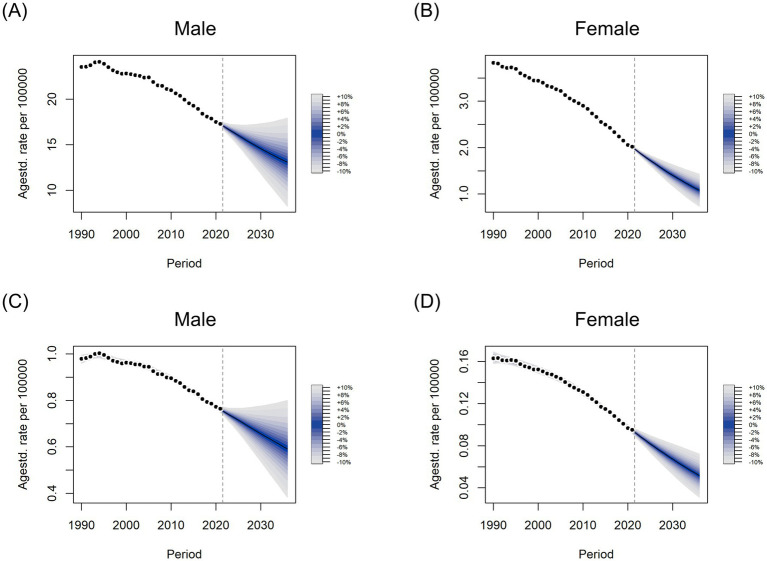
Predicted trends in age-standardized DALYs **(A,B)** and death **(C,D)** rates from 2022 to 2036 in males **(A,C)** and females **(B,D)** using Bayesian age-period-cohort (BAPC) models.

The DALYs and death rates for both sexes are projected to gradually decline from 2022 to 2036 (Figures 10A–D). The ASDALY in males is expected to decrease, reaching approximately 13.08 globally by 2036, while in females, it is projected to reach 1.08 (Figures 10A,B). Similarly, the ASDR for both sexes is anticipated to follow the same pattern as ASDALY (Figures 10C,D). Although these projections indicate a decline for both sexes, the burden of kidney cancer attributable to smoking will remain significantly higher in men than in women.

## Discussion

### Principal findings

Our study utilized data from the GBD 2021 database to summarize the global disease burden of kidney cancer attributable to smoking. Over the past 30 years, from 1990 to 2021, the global burden of smoking-induced kidney cancer has increased, with the number of death and DALYs rising by more than 50%. This rise can be attributed to aging populations, population growth, and advancements in diagnostic technologies, which have led to an increase in incidental kidney cancer diagnoses in many countries (20). Nevertheless, despite these increases, ASDR and ASDALY for smoking-associated kidney cancer have continued to decline globally, primarily due to significant advances in public health, including the implementation of tobacco control measures, particularly following the adoption of the Framework Convention on Tobacco Control (24). The decline in smoking rates has also been supported by heightened public awareness of the harmful effects of smoking and improvements in medical care. However, challenges in tobacco control remain.

Increasing evidence highlights the toxicity and carcinogenic effects of tobacco smoke on the kidneys, with nicotine being the primary addictive compound in tobacco (25). Previous studies have shown that long-term nicotine exposure may promote kidney cancer development by inducing abnormal proliferation and tumor transformation of renal epithelial cells, upregulating oxidative stress, and causing DNA damage (26–28). Additionally, other chemicals in tobacco smoke, such as carbon monoxide, polycyclic aromatic hydrocarbons, N-nitrosodiethylamine, and heavy metals like arsenic, lead, and cadmium are recognized human carcinogens (25, 29). Furthermore, a study in India demonstrated that long-term users of chewing tobacco had significantly increased levels of nicotine and cotinine (30).

Regarding gender differences in the risk of developing kidney cancer, a study on global cancer incidence statistics from 1978 to 2007 reported a 2:1 male-to-female incidence ratio, which remained consistent across age, year, and region (31). In terms of mortality, men account for nearly two-thirds of all deaths from RCC (32, 33). Similarly, our study reveals a significant gender disparity in the age-standardized rate of kidney cancer attributable to smoking, with rates in men more than eight times higher than in women. This can be linked to the higher prevalence of tobacco use among men; Since 1990, daily smoking rates have been approximately five times higher in men (25%) than in women (5.4%) (24). Women also tend to smoke for shorter durations and at lower intensities than men. However, existing evidence does not fully account for the observed sex differences in kidney cancer. While one analysis suggested that the survival advantage in premenopausal women may be linked to the estrogen axis (34), another study found that the sex ratio for kidney cancer remained stable across age groups, both before and after menopause (31), leaving the protective role of estrogen in cancer survival controversial. Biological differences, such as immune surveillance, genomic maintenance, and sex chromosomes, as well as lifestyle factors, could also contribute to the sex-related disparities in disease incidence.

Our findings reveal significant variations in the spatial distribution of the kidney cancer burden attributable to smoking across countries and territories. East Asia, Central Europe, and Eastern Europe bear the highest burden, while Oceania and Western Sub-Saharan Africa have the lowest. Regionally, East Asia had the highest absolute number of deaths and DALYs in 2021, with China, the world’s most populous country, leading in both metrics, followed by the United States. According to previous studies, China is the world’s largest producer and consumer of tobacco, with approximately 300 million smokers accounting for 40% of global cigarette consumption (35, 36). In recent years, China’s rapid economic growth and increasing wealth have contributed to rising smoking rates (37). Data from China between 2018 and 2019 indicated that half of smokers exhibit strong tobacco dependence, making quitting a significant challenge (38). Another reason is that, compared to other countries, China’s efforts to reduce or ban smoking in certain public places have been poorly coordinated and inadequately enforced, leading to increased population exposure to secondhand smoke (39). These factors may explain why China has the highest absolute number of smoking-attributable kidney cancer cases in the world.

Mongolia, also located in East Asia, has experienced the fastest increase in ASR of smoking-related kidney cancer between 1990 and 2021, which is concerning. This may be attributed to inadequate tobacco control measures and insufficient health education in the country. In 2009, 48% of Mongolian men aged 15 and older reportedly consumed tobacco, placing Mongolia among the countries with the highest prevalence of male smokers globally (40). A multi-stage random cluster sampling study in Mongolia revealed that a significant proportion of individuals with primary and secondary education believed that smoking at least one pack of cigarettes per day would be harmful to their health (41). Another national survey of schoolchildren in Mongolia revealed that the sale of cigarettes and single cigarettes to students near schools increased between 2014 and 2019 (42). Tobacco is a major risk factor for various diseases, not just kidney cancer. Consequently, large-scale tobacco control interventions, such as raising tobacco taxes, enhancing mass media campaigns, and strengthening school-based education, are urgently needed in Mongolia.

Sri Lanka experienced the largest decline in the ASR of smoking-attributable kidney cancer, likely due to the country’s strong tobacco control laws and legislation. The National Strategic Plan on Prevention and Control of Cancer in Sri Lanka (2020–2024), guided by the government, has developed a comprehensive strategy for tobacco control. Measures such as regulating tobacco product content, preventing exposure to tobacco smoke, controlling product disclosures, restricting advertising, managing the legal trade of tobacco products, and prohibiting sales to individuals under 21 have effectively reduced tobacco exposure and improved public health (43). However, the 17-month economic crisis in Sri Lanka, which began in late 2021, caused shortages of medicines and medical supplies, disrupting the treatment of various diseases, including cancer. This may affect cancer data beyond 2021 (44).

In 2021, the age group with the highest share of global kidney cancer deaths and DALYs attributable to smoking shifted five years older compared to 1990. Specifically, the highest number of global deaths for both men and women occurred in the 70–74 age group. According to the EAPC results, the ASR in the global population over 85 years old is rising, while the ASR in those under 80 is declining. This indicates that the burden of smoking-related kidney cancer is increasingly concentrated in older populations. This trend may be explained by the cumulative effects of smoking-related cancers, where prolonged smoking leads to the accumulation of carcinogens in the body. Moreover, older adults are at higher risk of comorbidities due to declining overall health and physiological functions (45). Advances in medical care, which have extended patient lifespans, further contribute to this shift. As a result, in the future, kidney cancer attributable to smoking is likely to cause more deaths among older individuals than younger ones. Strengthening early screening to reduce the discomfort and disability associated with advanced cancer and its treatments, while aiming to lower mortality, will pose a significant challenge.

Across SDI regions, from 1990 to 2021, the ASDR and ASDALY of smoking-attributable kidney cancer were generally higher in areas with higher SDI levels. A previous study examining the burden of kidney cancer morbidity and mortality from 1990 to 2019 found a strong positive association between kidney cancer burden and SDI, which aligns with our findings (20). One reason for the high incidence of kidney cancer in developed countries in recent decades may be advances in abdominal cross-sectional imaging technology, which have enabled the detection of even small kidney masses (46). It is estimated that up to 50% of the increase in kidney cancer incidence in developed countries is attributable to overdiagnosis (47).

However, as reflected in the EAPC values, smoking-induced kidney cancer ASDR and ASDALY in high and high-middle SDI areas have shown a sustained decline over the past 30 years. According to the 2015 global smoking prevalence study, the age-standardized prevalence of daily smoking decreased globally between 1990 and 2015 for both men and women, with the most significant reductions observed in high SDI countries and Latin America (24). This decline can be explained by the reduced use of unnecessary diagnostic methods in developed countries in recent years, along with increased tobacco taxes, which have led to a decrease in tobacco consumption (48, 49). Additionally, stricter regulations on smoking in public places, improved health awareness, and advances in medical treatment have further contributed to this trend. In low SDI regions, the prevalence of daily smoking among men and women is already low (24). However, despite the lower smoking rates, factors such as poor environmental conditions, low health awareness, and limited access to routine medical and diagnostic care have contributed to the ASDR and ASDALY remaining largely unchanged (50).

From 1990 to 2021, the SII increased from 8.11 to 12.08, indicating a growing disease burden in countries with high SDI. Over time, this gap widened, with the absolute difference in health burdens becoming more pronounced. In contrast, a negative CI value indicates that the disease burden is relatively concentrated in low SDI countries, highlighting the unequal distribution of the burden. However, the decline in CI (from −0.55 to −0.43) suggests that although low SDI countries continue to bear a greater share of the disease burden, the relative gap between high and low SDI groups is narrowing, signaling a reduction in relative inequality. When considering both the SII and CI, it becomes evident that while relative inequality is improving, the absolute differences in health burdens between groups are still increasing.

As a prediction model, the Bayesian APC model has been proven to be reliable. Therefore, we conducted a BAPC analysis to predict trends in age-standardized death and DALYs rates for smoking-related kidney cancer in both sexes. ASDR and ASDALY are projected to decline globally for both men and women from 2022 to 2036. However, the burden of smoking-related kidney cancer in men is expected to remain significantly higher than in women. It is important to note that increasing life expectancy and an aging society will likely exacerbate challenges for the older adult population. Thus, these projections should be interpreted with caution.

In summary, while the ASDR and ASDALY of smoking-related kidney cancer have decreased globally, significant disparities persist across regions, countries, and SDI levels. Although the disease burden is higher in countries with high SDI, it remains a serious issue in some developing countries, such as China, which has the highest absolute number of deaths, and Mongolia, which recorded the highest EAPC between 1990 and 2021. Health systems in these countries should implement measures to promote tobacco control, allocate health resources efficiently, and provide the necessary management for kidney cancer treatment. In addition, improved diagnostic methods and enhanced early detection techniques, such as identifying blood and urine markers, are crucial for achieving better outcomes for kidney cancer patients (5).

### Limitations

The study has several limitations. First, inadequate cancer registries in some low-income regions and countries have resulted in limited data quality, accuracy, and completeness regarding kidney cancer, which may introduce deviations in projections of disease burden trends. However, to improve data quality, we made efforts to standardize the research data. Second, since tobacco-use habits are self-reported, there is a potential for reporting bias across different countries and time periods. The scope of our study focused on smoking; however, due to limited data availability in the GBD 2021 dataset, other forms of tobacco use—such as chewing tobacco, secondhand smoke, and oral and nasal smokeless tobacco products, which have been used for centuries in many countries—were not included (17). No distinction was made between former and current smokers, the number of cigarettes smoked per day, or the duration of tobacco exposure. Third, our study focused solely on the burden of kidney cancer caused by tobacco use, without accounting for other confounding factors such as high BMI, exposure to trichloroethylene, and alcohol consumption (51). This omission may introduce some bias into the results.

## Conclusion

Between 1990 and 2021, both ASDR and ASDALY attributable to smoking in kidney cancer, which are positively correlated with SDI, have declined. However, significant demographic and geographic disparities persist, with the disease burden remaining higher in older populations and regions with elevated SDI levels. Moreover, while the overall burden is projected to decline annually over the next 15 years, it is expected to remain significantly higher in men. These findings emphasize the need for region-specific health prevention strategies to reduce smoking-related kidney cancer.

## Data Availability

The original contributions presented in the study are included in the article/Supplementary material, further inquiries can be directed to the corresponding author/s.
