# Supplementary material for: Global burden, trends, and disparities in kidney cancer attributable to smoking from 1990 to 2021
Source: Front Public Health. 2025 Jan 8;12:1506542. doi: 10.3389/fpubh.2024.1506542 (PMC11750797; doi:10.3389/fpubh.2024.1506542)
Supplement: Supplementary file 1 [file Supplementary_file_1.docx]

Global burden, trends, and disparities in kidney cancer attributable to smoking from 1990 to 2021

**Supplementary Information**

**Table S1.** Global burden of kidney cancer attributable to tobacco in 1990 and 2021 for both sex and all regions, with EAPC.

| Region | | | 1990 | | | | 2021 | | | | EAPC (1990–2021) | | |
| --- | --- | --- | --- | --- | --- | --- | --- | --- | --- | --- | --- | --- | --- |
|  | Deaths cases No. (95% UI) | ASDR per 100 000 No. (95% UI) | | DALYs No. (95% UI) | ASDALY per 100 000 No. (95% UI) | Deaths cases No. (95% UI) | | ASDR per 100 000 No. (95% UI) | DALYs No. (95% UI) | ASDALY per 100 000 No. (95% UI) | | ASDR No. (95% CI) | ASDALY No. (95% CI) |
| Global | 9,673 (6,072 to 13,617) | 0.25 (0.16 to 0.35) | | 251,336 (159,401 to 348,868) | 6.17 (3.9 to 8.61) | 16,216 (9,663 to 23,217) | | 0.19 (0.11 to 0.27) | 382,927 (233,635 to 536,755) | 4.37 (2.66 to 6.14) | | -0.93 (-1.01 to -0.86) | -1.15 (-1.23 to -1.06) |
| High SDI | 5,423 (3,350 to 7,710) | 0.49 (0.3 to 0.69) | | 136,388 (85,078 to 192,526) | 12.65 (7.91 to 17.8) | 7,046 (4,030 to 10,669) | | 0.33 (0.19 to 0.49) | 152,961 (89,126 to 226,095) | 7.68 (4.54 to 11.29) | | -1.34 (-1.45 to -1.22) | -1.64 (-1.77 to -1.50) |
| High-middle SDI | 2,963 (1,918 to 4,089) | 0.29 (0.19 to 0.41) | | 80,770 (52,628 to 110,173) | 7.78 (5.06 to 10.64) | 5,509 (3,390 to 7,688) | | 0.27 (0.17 to 0.38) | 138,425 (86,981 to 191,405) | 6.88 (4.32 to 9.5) | | -0.32 (-0.49 to -0.14) | -0.50 (-0.68 to -0.32) |
| Middle SDI | 942 (615 to 1,283) | 0.1 (0.06 to 0.13) | | 25,284 (16,536 to 34,605) | 2.35 (1.53 to 3.21) | 2,802 (1,718 to 3,871) | | 0.11 (0.06 to 0.15) | 70,248 (43,364 to 97,386) | 2.51 (1.55 to 3.48) | | 0.36 (0.29 to 0.43) | 0.33 (0.24 to 0.42) |
| Low-middle SDI | 282 (176 to 393) | 0.05 (0.03 to 0.07) | | 7,261 (4,549 to 10,079) | 1.17 (0.73 to 1.63) | 735 (455 to 1,050) | | 0.05 (0.03 to 0.08) | 18,224 (11,317 to 25,873) | 1.25 (0.77 to 1.77) | | 0.21 (0.16 to 0.26) | 0.18 (0.14 to 0.22) |
| Low SDI | 47 (27 to 68) | 0.02 (0.01 to 0.03) | | 1,194 (694 to 1,717) | 0.53 (0.31 to 0.77) | 101 (56 to 151) | | 0.02 (0.01 to 0.03) | 2,536 (1,407 to 3,762) | 0.5 (0.28 to 0.75) | | -0.29 (-0.37 to -0.22) | -0.35 (-0.42 to -0.27) |
| Andean Latin America | 9 (5 to 14) | 0.05 (0.03 to 0.07) | | 212 (123 to 319) | 1.06 (0.61 to 1.6) | 31 (17 to 51) | | 0.05 (0.03 to 0.09) | 666 (371 to 1,058) | 1.14 (0.63 to 1.82) | | 0.52 (0.38 to 0.66) | 0.35 (0.22 to 0.49) |
| Australasia | 89 (53 to 132) | 0.37 (0.22 to 0.55) | | 2,176 (1,319 to 3,190) | 9.35 (5.66 to 13.7) | 110 (58 to 181) | | 0.2 (0.11 to 0.33) | 2,403 (1,339 to 3,786) | 4.76 (2.69 to 7.4) | | -2.09 (-2.17 to -2.02) | -2.22 (-2.29 to -2.15) |
| Caribbean | 33 (20 to 50) | 0.13 (0.08 to 0.2) | | 838 (513 to 1,228) | 3.21 (1.96 to 4.71) | 67 (39 to 101) | | 0.12 (0.07 to 0.19) | 1,598 (931 to 2,361) | 2.95 (1.72 to 4.36) | | -0.15 (-0.26 to -0.03) | -0.25 (-0.37 to -0.13) |
| Central Asia | 75 (47 to 110) | 0.15 (0.09 to 0.22) | | 2,219 (1,402 to 3,211) | 4.38 (2.76 to 6.35) | 186 (119 to 262) | | 0.22 (0.14 to 0.31) | 5,299 (3,398 to 7,461) | 5.84 (3.75 to 8.25) | | 1.35 (0.99 to 1.71) | 1.03 (0.68 to 1.39) |
| Central Europe | 777 (500 to 1,087) | 0.51 (0.32 to 0.71) | | 21,030 (13,703 to 28,830) | 13.59 (8.84 to 18.64) | 1,091 (657 to 1,590) | | 0.49 (0.3 to 0.71) | 26,128 (16,114 to 37,549) | 12.35 (7.66 to 17.67) | | -0.05 (-0.27 to 0.17) | -0.24 (-0.48 to 0.01) |
| Central Latin America | 86 (51 to 124) | 0.11 (0.07 to 0.16) | | 2,068 (1,248 to 2,945) | 2.51 (1.51 to 3.6) | 186 (108 to 278) | | 0.08 (0.04 to 0.11) | 4,249 (2,514 to 6,243) | 1.69 (1 to 2.48) | | -1.38 (-1.50 to -1.26) | -1.46 (-1.57 to -1.34) |
| Central Sub-Saharan Africa | 3 (2 to 5) | 0.01 (0.01 to 0.03) | | 83 (44 to 148) | 0.36 (0.18 to 0.63) | 9 (4 to 17) | | 0.02 (0.01 to 0.03) | 256 (115 to 470) | 0.44 (0.19 to 0.8) | | 0.76 (0.42 to 1.09) | 0.83 (0.51 to 1.14) |
| East Asia | 989 (642 to 1,391) | 0.13 (0.08 to 0.18) | | 26,737 (17,317 to 37,593) | 2.98 (1.93 to 4.19) | 3,614 (2,156 to 5,143) | | 0.17 (0.1 to 0.23) | 90,535 (53,869 to 127,788) | 3.99 (2.37 to 5.65) | | 1.15 (0.98 to 1.32) | 1.27 (1.08 to 1.47) |
| Eastern Europe | 1,109 (732 to 1,540) | 0.38 (0.25 to 0.53) | | 32,358 (21,493 to 44,764) | 11.15 (7.41 to 15.47) | 1,659 (1,053 to 2,309) | | 0.46 (0.29 to 0.65) | 44,638 (28,604 to 61,835) | 12.81 (8.22 to 17.74) | | 0.31 (-0.02 to 0.64) | 0.08 (-0.27 to 0.42) |
| Eastern Sub-Saharan Africa | 14 (8 to 20) | 0.02 (0.01 to 0.03) | | 345 (195 to 509) | 0.47 (0.27 to 0.69) | 33 (16 to 51) | | 0.02 (0.01 to 0.03) | 823 (403 to 1,302) | 0.49 (0.25 to 0.77) | | 0.09 (0.01 to 0.17) | 0.11 (0.03 to 0.18) |
| High-income Asia Pacific | 543 (356 to 742) | 0.27 (0.18 to 0.37) | | 13,732 (9,110 to 18,509) | 6.59 (4.36 to 8.9) | 980 (574 to 1,443) | | 0.2 (0.12 to 0.28) | 18,364 (11,191 to 26,517) | 4.26 (2.63 to 6.08) | | -1.20 (-1.36 to -1.03) | -1.55 (-1.75 to -1.35) |
| High-income North America | 1,831 (1,090 to 2,629) | 0.54 (0.32 to 0.77) | | 48,457 (29,458 to 69,128) | 14.87 (9.09 to 21.13) | 2,365 (1,276 to 3,741) | | 0.35 (0.19 to 0.55) | 55,145 (31,522 to 84,516) | 8.66 (5.04 to 13.2) | | -1.46 (-1.63 to -1.28) | -1.86 (-2.05 to -1.68) |
| North Africa and Middle East | 198 (120 to 275) | 0.12 (0.07 to 0.17) | | 5,502 (3,399 to 7,546) | 3.07 (1.87 to 4.24) | 582 (360 to 849) | | 0.13 (0.08 to 0.2) | 15,605 (9,791 to 22,589) | 3.25 (2.03 to 4.76) | | 0.25 (0.20 to 0.29) | 0.14 (0.09 to 0.18) |
| Oceania | 1 (0 to 1) | 0.02 (0.01 to 0.04) | | 17 (8 to 27) | 0.56 (0.27 to 0.9) | 1 (1 to 2) | | 0.02 (0.01 to 0.03) | 40 (18 to 68) | 0.49 (0.23 to 0.84) | | -0.64 (-0.76 to -0.52) | -0.59 (-0.71 to -0.47) |
| South Asia | 213 (133 to 298) | 0.04 (0.03 to 0.06) | | 5,365 (3,351 to 7,485) | 0.94 (0.59 to 1.32) | 528 (329 to 739) | | 0.04 (0.02 to 0.05) | 12,512 (7,705 to 17,580) | 0.84 (0.52 to 1.19) | | -0.51 (-0.58 to -0.44) | -0.56 (-0.62 to -0.50) |
| Southeast Asia | 172 (107 to 242) | 0.07 (0.05 to 0.1) | | 4,524 (2,800 to 6,352) | 1.73 (1.07 to 2.44) | 487 (310 to 685) | | 0.08 (0.05 to 0.11) | 12,609 (7,990 to 17,738) | 1.83 (1.16 to 2.57) | | -0.07 (-0.18 to 0.04) | -0.05 (-0.16 to 0.05) |
| Southern Latin America | 187 (112 to 270) | 0.4 (0.24 to 0.58) | | 5,167 (3,199 to 7,313) | 10.94 (6.78 to 15.49) | 345 (195 to 525) | | 0.4 (0.22 to 0.6) | 8,732 (5,156 to 12,825) | 10.25 (6.06 to 14.98) | | 0.10 (-0.12 to 0.33) | -0.12 (-0.33 to 0.08) |
| Southern Sub-Saharan Africa | 21 (11 to 31) | 0.08 (0.04 to 0.12) | | 539 (298 to 809) | 1.94 (1.06 to 2.92) | 34 (20 to 47) | | 0.06 (0.04 to 0.09) | 890 (543 to 1,247) | 1.47 (0.9 to 2.06) | | -0.86 (-0.96 to -0.75) | -0.79 (-0.90 to -0.69) |
| Tropical Latin America | 177 (109 to 252) | 0.2 (0.12 to 0.28) | | 4,712 (2,937 to 6,648) | 4.95 (3.06 to 7.02) | 445 (248 to 686) | | 0.17 (0.1 to 0.27) | 10,553 (6,022 to 15,842) | 4.03 (2.3 to 6.06) | | -0.50 (-0.58 to -0.42) | -0.79 (-0.88 to -0.70) |
| Western Europe | 3,140 (1,936 to 4,437) | 0.54 (0.33 to 0.76) | | 75,114 (46,895 to 105,381) | 13.56 (8.5 to 19.02) | 3,447 (1,972 to 5,219) | | 0.36 (0.21 to 0.53) | 71,438 (41,622 to 104,623) | 8.25 (4.85 to 11.9) | | -1.26 (-1.33 to -1.19) | -1.51 (-1.60 to -1.41) |
| Western Sub-Saharan Africa | 6 (3 to 9) | 0.01 (0 to 0.01) | | 141 (80 to 215) | 0.16 (0.09 to 0.24) | 17 (10 to 26) | | 0.01 (0.01 to 0.01) | 444 (256 to 681) | 0.22 (0.12 to 0.34) | | 1.07 (1.01 to 1.13) | 1.10 (1.03 to 1.17) |

**Table S2.** Estimated Annual percentage change in mortality rate between 1990 and 2021 by age group and SDI region.

| Region | Age Group | | | | | | | | | | | | | |
| --- | --- | --- | --- | --- | --- | --- | --- | --- | --- | --- | --- | --- | --- | --- |
|  | 30-34 years | 35-39 years | 40-44 years | 45-49 years | 50-54 years | 55-59 years | 60-64 years | 65-69 years | 70-74 years | 75-79 years | 80-84 years | 85-89 years | 90-94 years | 95+ years |
| Global | -1.65 (-1.79,-1.50) | -2.11 (-2.20,-2.01) | -2.38 (-2.55,-2.21) | -2.11 (-2.44,-1.79) | -1.80 (-2.08,-1.53) | -1.35 (-1.55,-1.15) | -1.28 (-1.37,-1.19) | -1.34 (-1.42,-1.25) | -1.00 (-1.15,-0.85) | -0.57 (-0.73,-0.41) | -0.13 (-0.28,0.02) | 0.44 (0.30,0.59) | 0.71 (0.62,0.80) | 0.54 (0.46,0.63) |
| High SDI | -2.61 (-2.78,-2.45) | -3.17 (-3.33,-3.01) | -3.48 (-3.72,-3.25) | -3.14 (-3.45,-2.82) | -2.51 (-2.77,-2.24) | -2.00 (-2.18,-1.81) | -1.93 (-2.04,-1.83) | -1.67 (-1.77,-1.57) | -1.31 (-1.45,-1.17) | -0.74 (-0.88,-0.60) | -0.01 (-0.13,0.12) | 0.67 (0.55,0.79) | 0.90 (0.84,0.96) | 0.76 (0.65,0.87) |
| High-middle SDI | -0.41 (-0.68,-0.13) | -0.93 (-1.09,-0.78) | -1.44 (-1.64,-1.24) | -1.35 (-1.74,-0.96) | -1.22 (-1.54,-0.90) | -0.79 (-1.05,-0.54) | -0.48 (-0.62,-0.34) | -0.60 (-0.81,-0.40) | -0.06 (-0.32,0.20) | 0.37 (0.13,0.60) | 0.58 (0.39,0.77) | 0.82 (0.61,1.02) | 0.88 (0.70,1.07) | 0.62 (0.45,0.78) |
| Middle SDI | -0.26 (-0.49,-0.03) | -0.43 (-0.70,-0.17) | 0.01 (-0.25,0.26) | 0.27 (0.01,0.54) | 0.12 (0.01,0.22) | 0.22 (0.10,0.35) | 0.16 (0.07,0.25) | 0.21 (0.14,0.28) | 0.37 (0.29,0.45) | 0.83 (0.70,0.96) | 0.64 (0.55,0.74) | 0.25 (0.07,0.43) | 0.49 (0.32,0.67) | 0.22 (0.00,0.45) |
| Low-middle SDI | -0.36 (-0.44,-0.29) | -0.54 (-0.65,-0.42) | -0.38 (-0.50,-0.25) | -0.15 (-0.34,0.04) | -0.10 (-0.21,0.02) | 0.42 (0.38,0.46) | 0.23 (0.19,0.27) | 0.05 (0.01,0.09) | 0.12 (0.04,0.19) | 0.14 (0.06,0.22) | 0.38 (0.29,0.48) | 0.60 (0.47,0.73) | 0.63 (0.46,0.81) | 0.72 (0.50,0.94) |
| Low SDI | 0.39 (0.06,0.73) | 0.14 (-0.05,0.33) | -0.01 (-0.05,0.02) | -0.20 (-0.29,-0.11) | -0.53 (-0.65,-0.41) | -0.39 (-0.48,-0.31) | -0.52 (-0.60,-0.44) | -0.57 (-0.65,-0.49) | -0.22 (-0.31,-0.13) | -0.24 (-0.32,-0.15) | -0.07 (-0.15,0.01) | 0.03 (-0.14,0.21) | 0.27 (-0.02,0.56) | 0.45 (0.15,0.74) |

**Table S3.** Estimated Annual percentage change in DALYs rate between 1990 and 2021 by age group and SDI region.

| Region | | Age Group | | | | | | | | | | | | | |  |
| --- | --- | --- | --- | --- | --- | --- | --- | --- | --- | --- | --- | --- | --- | --- | --- | --- |
|  | 30-34 years | | 35-39 years | 40-44 years | 45-49 years | 50-54 years | 55-59 years | 60-64 years | 65-69 years | 70-74 years | 75-79 years | 80-84 years | 85-89 years | 90-94 years | 95+ years | |
| Global | -1.60 (-1.75,-1.46) | | -2.06 (-2.15,-1.97) | -2.34 (-2.51,-2.17) | -2.08 (-2.40,-1.75) | -1.76 (-2.04,-1.48) | -1.30 (-1.50,-1.10) | -1.23 (-1.33,-1.14) | -1.29 (-1.37,-1.20) | -0.96 (-1.11,-0.81) | -0.54 (-0.70,-0.38) | -0.12 (-0.27,0.04) | 0.44 (0.30,0.59) | 0.71 (0.62,0.80) | 0.49 (0.40,0.57) | |
| High SDI | -2.54 (-2.71,-2.37) | | -3.10 (-3.26,-2.94) | -3.41 (-3.65,-3.17) | -3.07 (-3.39,-2.75) | -2.44 (-2.71,-2.17) | -1.93 (-2.11,-1.74) | -1.86 (-1.97,-1.75) | -1.60 (-1.70,-1.50) | -1.25 (-1.39,-1.11) | -0.69 (-0.83,-0.55) | 0.02 (-0.11,0.14) | 0.67 (0.56,0.79) | 0.90 (0.84,0.97) | 0.69 (0.58,0.81) | |
| High-middle SDI | -0.34 (-0.62,-0.06) | | -0.86 (-1.01,-0.70) | -1.37 (-1.56,-1.17) | -1.29 (-1.68,-0.91) | -1.16 (-1.47,-0.84) | -0.74 (-1.00,-0.49) | -0.43 (-0.56,-0.29) | -0.56 (-0.76,-0.36) | -0.02 (-0.27,0.23) | 0.39 (0.16,0.63) | 0.59 (0.40,0.79) | 0.82 (0.62,1.01) | 0.89 (0.70,1.07) | 0.59 (0.44,0.75) | |
| Middle SDI | -0.20 (-0.43,0.03) | | -0.37 (-0.63,-0.10) | 0.07 (-0.18,0.33) | 0.33 (0.06,0.60) | 0.17 (0.07,0.28) | 0.27 (0.15,0.39) | 0.20 (0.12,0.29) | 0.26 (0.19,0.33) | 0.41 (0.33,0.48) | 0.85 (0.72,0.98) | 0.65 (0.56,0.75) | 0.25 (0.07,0.42) | 0.49 (0.32,0.67) | 0.16 (-0.06,0.39) | |
| Low-middle SDI | -0.34 (-0.42,-0.27) | | -0.51 (-0.62,-0.40) | -0.35 (-0.47,-0.23) | -0.13 (-0.32,0.06) | -0.08 (-0.20,0.04) | 0.44 (0.40,0.48) | 0.25 (0.21,0.29) | 0.07 (0.03,0.11) | 0.13 (0.06,0.21) | 0.15 (0.06,0.23) | 0.39 (0.29,0.48) | 0.60 (0.47,0.73) | 0.63 (0.46,0.81) | 0.69 (0.49,0.90) | |
| Low SDI | 0.41 (0.08,0.74) | | 0.16 (-0.03,0.35) | 0.01 (-0.02,0.04) | -0.18 (-0.27,-0.08) | -0.52 (-0.64,-0.40) | -0.38 (-0.46,-0.29) | -0.51 (-0.59,-0.43) | -0.57 (-0.65,-0.48) | -0.22 (-0.31,-0.13) | -0.24 (-0.32,-0.15) | -0.07 (-0.15,0.00) | 0.03 (-0.15,0.20) | 0.27 (-0.02,0.55) | 0.46 (0.17,0.75) | |
